# Preparation and Enhanced Catalytic Performance of a Polyhedral BiVO_4_-Nanoparticle-Modified ZnO Flower-like Nanorod Structure Composite Material

**DOI:** 10.3390/nano15191536

**Published:** 2025-10-09

**Authors:** Yuanyuan Lv, Neng Li, Jin Liu, Quanhui Liu, Xueqi Hui, Qiang Li

**Affiliations:** 1 School of Communication and Information Engineering, Xi’an University of Science and Technology, Xi’an 710054, China; 23207035001@stu.xust.edu.cn (N.L.); liujin@xust.edu.cn (J.L.); 2 Shaanxi Yanchang Petroleum Yulin Kekegai Coal Industry Co., Ltd., Yulin 719000, China

**Keywords:** hydrothermal method, BiVO_4_@ZnO, heterojunction composites, photocatalytic degradation, methylene blue dyes

## Abstract

Organic pollutants pose a significant threat to both the ecological environment and human health. In this study, BiVO_4_@ZnO heterojunction composites were synthesized via a two-step hydrothermal method. The incorporation of polyhedral BiVO_4_ onto the flower-like structure of ZnO effectively enhanced the photocatalytic performance of the composite. Compared with ZnO flower-like nanorods, the BiVO_4_@ZnO heterojunction composite photocatalysts achieved degradation efficiencies of 93.18% (k = 0.09063) and 89.64% (k = 0.007661) for methylene blue (MB) within 30 min under ultraviolet and visible light irradiation, respectively. The photocatalytic activity of the BiVO_4_@ZnO composites was also evaluated against various organic dyes, including rhodamine B (RhB), Congo red (CR), methyl orange (MO), and methylene blue (MB). Under ultraviolet light, the catalysts showed particularly high activity toward MB and CR. The enhanced photocatalytic performance can be attributed to two main factors: firstly, the heterojunction facilitates the separation of photogenerated electron-hole pairs, thereby improving photocatalytic efficiency; secondly, the composite exhibits a broadened and enhanced light absorption range. Furthermore, the BiVO_4_@ZnO heterojunction composites demonstrate excellent cyclic catalytic stability and structural integrity. This study offers a clean and efficient strategy for the photocatalytic degradation of aqueous organic pollutants.

## 1. Introduction

In recent years, the release of pollutants such as organic dyes and antibiotics has exacerbated environmental degradation. These pollutants, with their complex structures and high solubility in water, have seriously affected our health and survival [1,2]. In response, photocatalytic technology has become a hot topic due to its potential in environmental protection and energy conversion [3]. This technology harnesses solar energy to drive chemical reactions that can break down organic pollutants and produce hydrogen gas, which holds great practical significance [4,5].

However, traditional photocatalytic materials, especially ZnO-based photocatalysts, still face significant challenges when it comes to efficiency and stability [6,7]. ZnO is a widely studied semiconductor material renowned for its excellent photocatalytic performance, low cost, and eco-friendliness. However, its wide bandgap (~3.37 eV) restricts its absorption primarily to the ultraviolet region, limiting its utilization of visible light. This leads to a high recombination rate of photogenerated charge carriers and unstable photocatalytic activity, which severely hinders its effectiveness in practical applications [8,9]. Furthermore, prolonged light exposure can induce the photocorrosion of ZnO particles, further degrading their catalytic performance [10]. Nevertheless, these very limitations also make ZnO a highly adaptable platform for modification, offering opportunities for significant property enhancements [11,12].

To enhance the photocatalytic performance of ZnO, researchers have explored various modifications strategies, including morphology control [13,14], elemental doping [15,16], deposition of noble metals [17,18], and the construction of heterojunctions [19,20,21,22]. Due to their enhanced light harvesting capability, optimized charge separation effect upon light induction, and a facile preparation process, the construction of heterostructures based on ZnO has produced outstanding photocatalytic activity [23,24]. Recently, analysis of charge transfer in these heterostructures has revealed the significant advantages of a well-designed stepwise (S-Scheme) mechanism, leading to efficient redox reactions [25]. BiVO_4_, which excels at absorbing visible light, has become a top choice for combining with ZnO because of its moderate bandgap of about 2.4 eV. When ZnO is paired with BiVO_4_, it can reduce the recombination of photo-generated carriers and improve photocatalytic efficiency [26,27,28]. Moreover, ZnO unique flower-like structure provides an excellent base for growing BiVO_4_, enhancing the combined effect of the two materials [29,30]. Therefore, preparing BiVO_4_@ZnO heterojunction composites, especially using polyhedral BiVO_4_ nanoparticles to modify ZnO flower-like structures, can significantly improve catalytic performance. The high specific surface area and numerous active sites of polyhedral BiVO_4_ nanoparticles provide more spots for photocatalytic reactions, while the ZnO nanorod-like structure can enhance the material’s ability to capture light and conduct electricity. This composite material helps separate and move photo-generated charge carriers, improves light utilization, and significantly boosts photocatalytic activity and stability.

This study aims to address the limitation of efficiency in single-component photocatalysts. Innovatively, a polyhedral BiVO_4_ nanoparticle-modified ZnO flower-like microrod composites was constructed, moving beyond simple material mixing. By leveraging morphology control and interface engineering, an efficient composite photocatalytic system was developed. The innovation is demonstrated by utilizing the polyhedral BiVO_4_ to expand the light response range and provide abundant reaction sites, while employing the hierarchical flower-like structure of zinc oxide to facilitate charge separation and transport. The synergistic effect between the two components collectively significantly enhances the photocatalytic performance.

## 2. Experiment

This experiment adopts a two-step hydrothermal method for preparing BiVO_4_@ZnO Composite nanomaterials.

### 2.1. Preparation of ZnO Flower-like Nanorods

First, 20 mL of 0.05 mol/L zinc acetate solution was added dropwise into 20 mL of a 0.7 mol/L sodium hydroxide solution under magnetic stirring. The mixture was stirred for 30 min to form a homogeneous precursor solution, resulting in a molar ratio of zinc acetate to sodium hydroxide of 1:14. The resulting clear and uniform precursor solution was then transferred into a 50 mL stainless steel autoclave and heated in an oven at 100 °C for 10 h. After the reaction was complete, the system was cooled to room temperature. The product was collected and washed three times with deionized water and absolute ethanol. Finally, it was dried in a vacuum oven at 60 °C for 12 h to obtain ZnO flower-like nanorod structures.

### 2.2. Preparation of BiVO_4_@ZnO Heterojunction Composites

In a typical process, 0.3 mmol/L of Bismuth nitrate pentahydrate (Bi(NO_3_)_3_·5H_2_O) and 2 mmol/L of Sodium orthovanadate dodecahydrate (Na_3_VO_4_·12H_2_O) were separately placed in deionized water. After 10 min of ultrasonic treatment, stirring was continued on a magnetic stirrer for another 30 min. Subsequently, the Bi(NO_3_)_3_·5H_2_O solution was added dropwise to the Na_3_VO_4_·12H_2_O solution, and stirring was further maintained for 30 min to obtain a yellow suspension (70 mL), which served as the precursor solution. Then, 0.035 mol/L of the prepared ZnO flower-like nanorods were added to the aforementioned precursor solution, followed by 30 min of stirring to form a homogeneous mixed solution. The resulting mixture was transferred into a 100 mL Teflon-lined steel autoclave and maintained at 160 °C for 6 h. After the reaction, the system was cooled to room temperature. The product was collected, washed three times with deionized water and absolute ethanol respectively, and then dried in a vacuum drying oven at 60 °C for 12 h to obtain the BiVO_4_@ZnO heterojunction composites.

### 2.3. Characterization

XRD (XRD, 6100, SHIMADZU, Kyoto, Japan) technology was utilized to determine the crystal structure and purity of the material. The morphology of the material was observed using scanning electron microscopy (ZEISS-GeminiSEM 360, Jena, Germany). Surface EDS and element distribution of the prepared samples were characterized via SEM-EDX and Mapping analysis. Chemical states and interactions of each element within the material were analyzed using X-ray photoelectron spectroscopy (Thermo Fisher-Nexsa, Waltham, MA, USA) test results. The functional groups and compound structures of composite materials were studied using an FTIR spectrometer (Shimadzu-IRTracer 100, Kyoto, Japan). The UV-VIS diffuse reflectance spectroscopy (UV-VIS DRS) test was conducted on the ZnO flower-like nanorods and BiVO_4_@ZnO heterojunction composite materials using an ultraviolet–visible–near-infrared spectrophotometer (Perkin Elmer-Lambda 750(s), Hopkinton, MA, USA). Nitrogen adsorption/desorption isotherm measurements were conducted on the samples at 77K using a Quantachrome instrument (Micromeritics-ASAP 2460, Norcross, GA, USA).

### 2.4. Evaluation of Photocatalytic Performance of MB, RhB, MO, and CR Dyes

The photocatalytic performance of ZnO flower-like nanorods and BiVO_4_@ZnO heterojunction composites was tested using a self-built photocatalytic reaction system, which was equipped with an 18 W ultraviolet mercury lamp with a main wavelength of 365 nm and a 20 W incandescent lamp as the visible light source, located 10 cm away from the catalytic reagent. Four typical organic dyes, namely, methylene blue (MB), rhodamine B (RhB), methyl orange (MO) and Congo red (CR), were selected as the target pollutants. Then, 20 mg of the ZnO flower-like nanorods and BiVO_4_@ZnO heterojunction composite catalysts was added to 50 mL of dye solutions with an initial concentration of 20 mg/L for each. The mixture was magnetically stirred at a speed of 200 r/min for 10 min in a dark room environment. After the system reached adsorption–desorption equilibrium, it was placed under the ultraviolet and visible light sources respectively for the photocatalytic degradation reaction. During the reaction, 4 mL of the reaction solution was taken from the reactor every 10 min. The catalyst was separated by centrifugation at 8000 r/min for 5 min using a tabletop high-speed centrifuge. The supernatant was taken to determine the absorbance of the dye at the maximum absorption wavelength by using a UV-Vis spectrophotometer (Hitachi Ltd.U-3310, Tokyo, Japan). The degradation concentration of the dye was calculated according to the Lambert–Beer law. The photocatalytic experiments for the other three dyes all strictly followed the above operation procedures. All the sampled samples were hermetically stored in the dark room environment.

## 3. Results and Discussion

**Figure 1:**
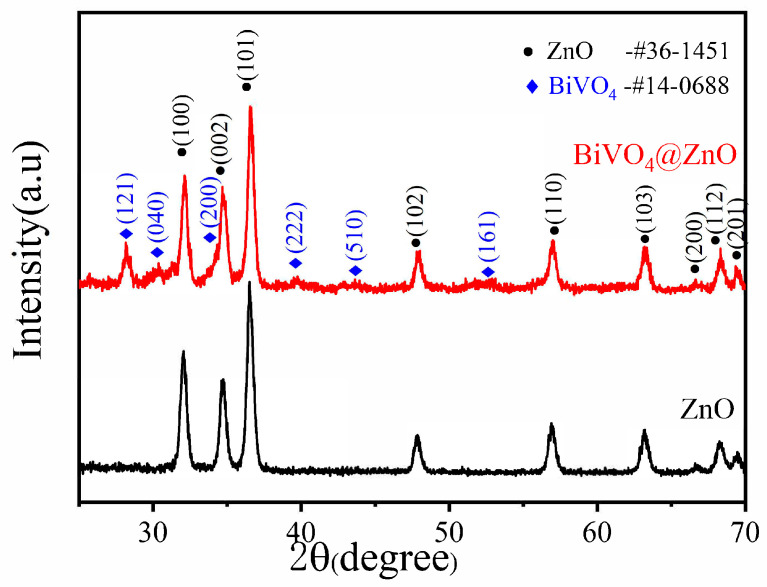
XRD pattern of ZnO and BiVO_4_@ZnO heterojunction composites.

The crystal structure of the ZnO and BiVO_4_@ZnO heterojunction composites was characterized using XRD, and the resulting patterns are presented in Figure 1. As shown in the figure, the diffraction peaks marked with circles correspond to those of the standard hexagonal wurtzite structure of ZnO (JCPDS No. 36-1451), namely, the (100), (002), (101), (102), (110), (103), (200), and (112) planes. This confirms that the synthesized ZnO possesses a pure hexagonal wurtzite crystal structure. Furthermore, the peaks marked with diamonds at 2θ angles of 28.8°, 30.06°, 35°, 40°, and 54.2° match the standard diffraction pattern for polyhedral BiVO_4_ (JCPDS No. 14-0688), corresponding to the (121), (040), (200), (222), and (161) planes, respectively. This confirms the successful formation of a composite containing both ZnO and BiVO_4_. Additionally, the absence of any extraneous diffraction peaks suggests the composite contains only ZnO and BiVO_4_, indicating high purity.

**Figure 2:**
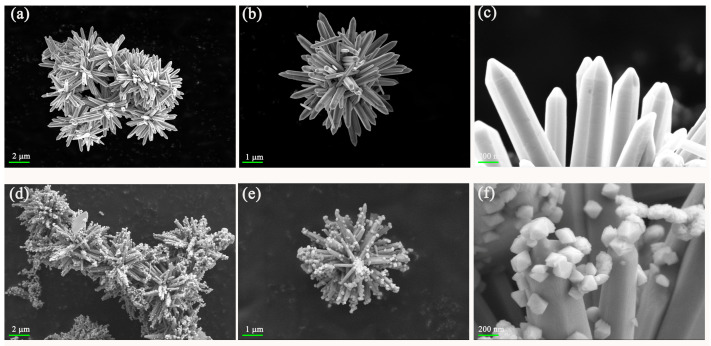
SEM image of (**a**–**c**) ZnO and (**d**–**f**) BiVO_4_@ZnO heterojunction composites.

The microstructure and morphology of the ZnO and BiVO_4_@ZnO heterojunction composites were characterized of the pure ZnO, revealing a densely packed bouquet-like microstructure composed of clustered ZnO nanorods. This clustered arrangement increases the specific surface area of the materials, which is beneficial for enhancing photocatalytic efficiency by providing more active sites. Figure 2c presents a high-magnification detail image of ZnO nanorods, confirming their distinct rod-like morphology with protruding tips, a close-packed arrangement, and a relatively smooth surface. Figure 2d,e depict the microstructure of the BiVO_4_@ZnO heterojunction composites, showing that BiVO_4_ nanoparticles are distributed on the flower-like ZnO structure, uniformly coating the surface of the ZnO nanorods. Figure 2f provides a high-magnification image of the composite materials, clearly revealing polyhedral BiVO_4_ nanoparticles adhered to the smooth surface of the ZnO nanorods. This nanoparticle-decorated nanorod structure is instrumental in facilitating the separation of photogenerated electrons and holes, thereby enhancing the photocatalytic efficacy.

**Figure 3:**
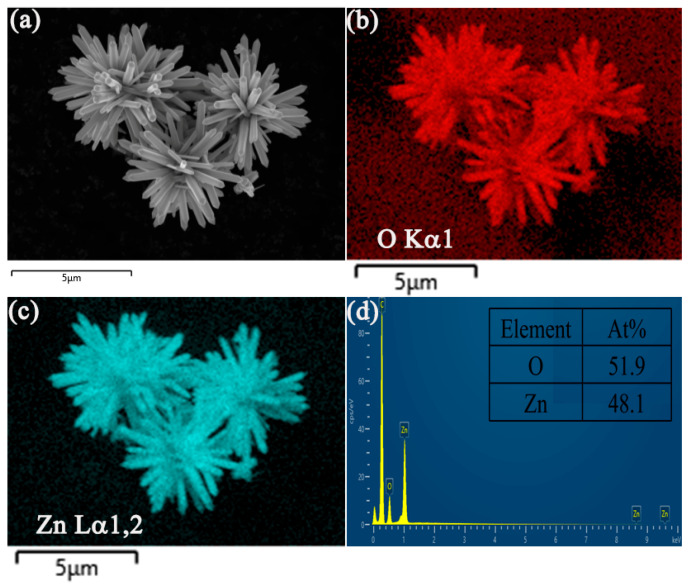
Mapping Diagram(**a**–**c**) and EDS (**d**) of pure ZnO flower-like nanorods.

The surface composition and elemental distribution of the synthesized ZnO nanomaterials were examined using energy-dispersive X-ray spectroscopy (EDS) coupled with scanning electron microscopy (SEM-EDS) and elemental mapping analysis, as depicted in Figure 3. Figure 3a presents the SEM image of the ZnO nanomaterials, revealing a densely packed, flower-like structure. In Figure 3b,c, the elements Zn and O are visualized in flower-like patterns with varying colors. The uniform color intensity suggests that these elements are homogeneously distributed throughout the materials. The absence of other elements in the elemental map indicates high sample purity. Furthermore, the energy-dispersive X-ray spectroscopy analysis shown in Figure 3d reveals that the sample contains 51.9% oxygen and 48.1% zinc by weight, with a ratio close to 1:1, which is consistent with the mapping analysis results.

**Figure 4:**
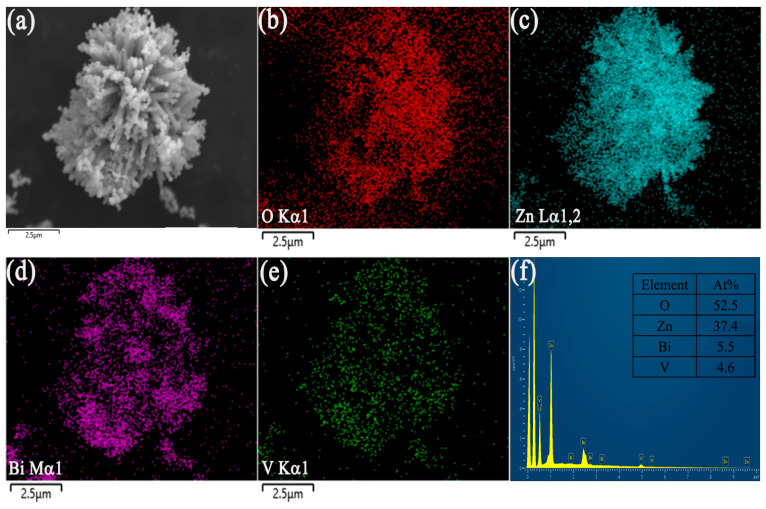
Mapping Diagram(**a**–**e**) and EDS (**f**) of the BiVO_4_@ZnO heterojunction composites.

The surface composition and elemental distribution of the BiVO_4_@ZnO heterojunction composites were analyzed using SEM-EDX and mapping analysis, as displayed in Figure 4. Figure 4a shows an SEM image of the BiVO_4_@ZnO heterojunction composites, showcasing its flower-shaped cluster structure. The elemental maps for O, Zn, Bi, and V are presented in Figure 4b–e, respectively. The intensity of the color in these maps corresponds to the elemental content. The O element exhibits the highest signal intensity, suggesting its highest concentration, followed by Zn. In contrast, the signals for V and Bi are lighter, indicating their lower concentrations. No other elements were detected, confirming the sample’s high chemical purity. The EDS spectrum in Figure 4f provides quantitative results, showing the sample contains 52.5% O, 37.4% Zn, 5.5% Bi, and 4.6% V by percentage. These quantitative results agree well with the qualitative mapping analysis.

**Figure 5:**
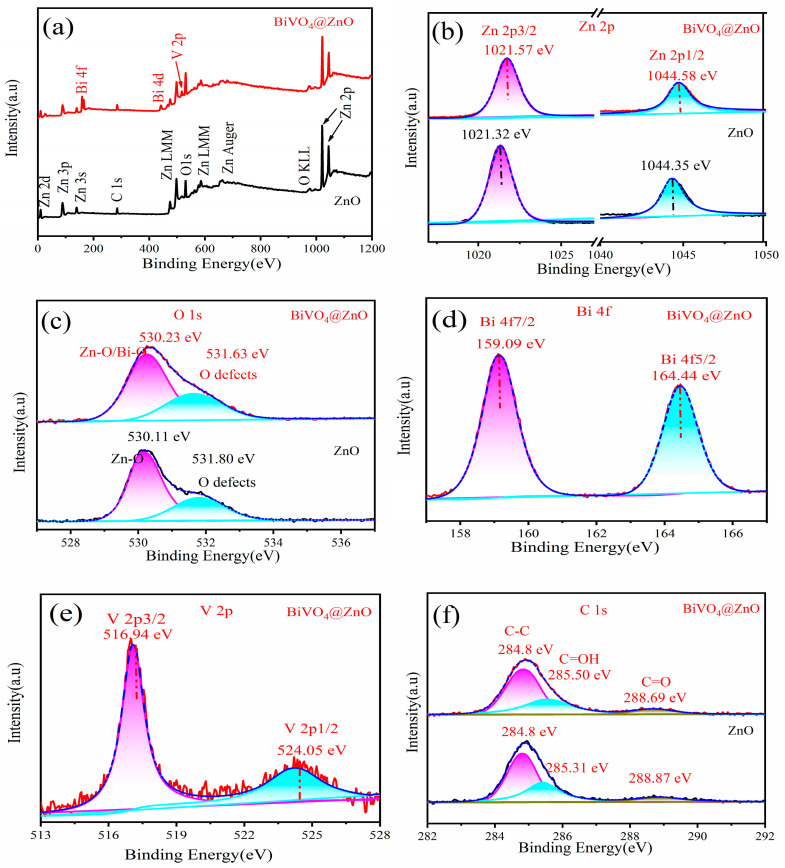
X-ray-photoelectron-spectroscopy spectra of obtained pure ZnO flower-like nanorods and BiVO_4_@ZnO heterojunction composites: (**a**) Full spectra of the samples, (**b**–**f**) binding states of Zn 2p, O 1s, Bi 4f, V 2p and C 1s, respectively.

To investigate the elemental composition, chemical states, and surface interactions of pure ZnO flower-like nanorods and BiVO_4_@ZnO heterojunction composites, X-ray photoelectron spectroscopy (XPS) measurements were performed, with the results summarized in Figure 5. Figure 5a presents the XPS survey spectra of both samples. The pure ZnO nanorods show characteristic peaks of Zn and O, while the BiVO_4_@ZnO heterojunction composites exhibit additional peaks corresponding to Bi and V, confirming the successful incorporation of BiVO_4_ onto ZnO. The high-resolution Zn 2p spectra are displayed in Figure 5b. For pure ZnO, the peaks located at 1021.32 eV and 1044.35 eV are assigned to Zn 2p3/2 and Zn 2p1/2, respectively. In the BiVO_4_@ZnO heterojunction composites, these peaks shift slightly to higher binding energies of 1021.57 eV and 1044.58 eV [31,32]. The spin-orbit splitting value of 23.00 eV in both samples indicates that Zn predominantly exists in the Zn^2+^ state [33,34]. The positive shift in binding energy is attributed to the formation of a Type-II heterojunction between ZnO and BiVO_4_, which promotes the transfer of photogenerated electrons from the conduction band of ZnO to that of BiVO_4_. This reduces the electron density around Zn atoms, increasing the binding energy, while the resulting built-in electric field enhances charge separation.

As shown in Figure 5c, the O 1s spectrum of flower-like nanorods is fitted with two components: a main peak at 530.11 eV (attributed to Zn-O bonds) and a shoulder at 531.80 eV associated with oxygen vacancies [35]. In the BiVO_4_@ZnO heterojunction composites, the main peak shifts to 530.23 eV, which can be ascribed to the synergistic interaction between Zn-O and Bi-O bonds, indicating an altered chemical environment at the heterointerface [36]. Concurrently, the oxygen-vacancy-related peak shifts to 531.63 eV, suggesting that the incorporation of BiVO_4_ modulates both the chemical bonding environment and the distribution of oxygen defects. These changes reflect interfacial charge redistribution, further corroborating the role of heterojunction-induced electronic modulation in promoting the separation and transport of photogenerated carriers, thereby enhancing photocatalytic performance. Figure 5d shows the high-resolution Bi 4f spectrum of the BiVO_4_@ZnO heterojunction composites, with peaks observed at 159.09 eV (Bi 4f7/2) and 164.44 eV (Bi 4f5/2), confirming the presence of Bi^3+^ [37]. The V 2p spectrum (Figure 5e) displays signals at 516.94 eV (V 2p3/2) and 524.05 eV (V 2p1/2), indicating that V is in the +5 oxidation state, consistent with the typical electronic environment in BiVO_4_ [38]. Figure 5f compares the C 1s spectra of pure ZnO and the BiVO_4_@ZnO heterojunction composites. Three constituent peaks are identified at approximately 284.8 eV, 285.5 eV (285.3 eV in the composite), and 288.7 eV (288.9 eV in the composite), corresponding to sp2 hybridized carbon (C-C/C=C), C-OH, and C=O functional groups, respectively. The slight shifts in binding energy support the occurrence of surface electronic restructuring due to charge transfer across the heterojunction interface, in agreement with the proposed band alignment mechanism [39].

**Figure 6:**
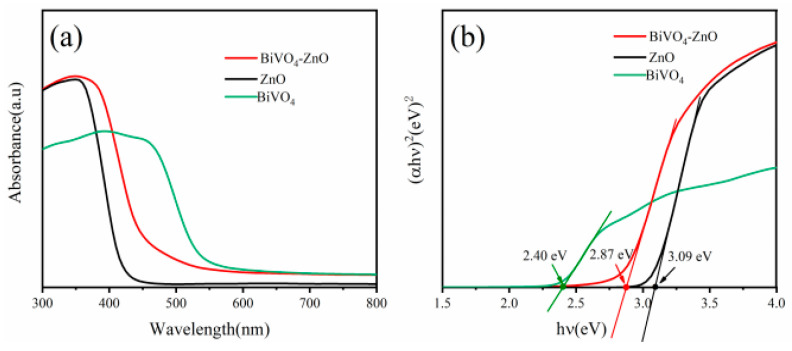
(**a**) UV–Vis absorption spectrum and (**b**) the (αhν)^2^ versus (hν) plots of pure ZnO flower-like nanorods, BiVO_4_@ZnO heterojunction composites and pure BiVO_4_ materials.

Figure 6 shows the UV–visible absorption spectra and the corresponding (αhν)^2^ versus (hν) plots for the pure ZnO flower-like nanorods, BiVO_4_@ZnO heterojunction composites and pure BiVO_4_ materials. As shown in Figure 6a, the absorption edge of the pure ZnO nanorods is located at approximately 380 nm, with absorption occurring primarily in the ultraviolet region, which is characteristic of its wide bandgap. In contrast, pure BiVO_4_ exhibits a red-shifted absorption edge around 520 nm, indicating a broader visible-light absorption range due to its narrower band gap.The BiVO_4_@ZnO heterojunction composites demonstrate significantly enhanced absorption, extending from the ultraviolet to the visible region (up to ~450 nm). This broadened absorption is attributed to the synergistic interaction between BiVO_4_ and ZnO, as well as interfacial effects within the composite, which collectively contribute to its improved photocatalytic performance.

The curve of the photon energy for semiconductor nanomaterials can be calculated using the following equation [40]:

$$\alpha h\nu =A{\left(h\nu -Eg\right)}^{n}$$

In this context, the variables *α*, *h*, *v*, *A*, and *Eg* represent the absorption coefficient, Planck’s constant, optical frequency, proportionality constant, and band gap energy, respectively. The value of n is determined by the characteristics of the semiconductor material, with n = 1/2 for direct band gap semiconductors and n = 2 for indirect band gap semiconductors. As depicted in Figure 6b, the band gaps of the pure ZnO flower-like nanorods, BiVO_4_@ZnO heterojunction composites, and pure BiVO_4_ are measured to be 3.09 eV, 2.87 eV, and 2.40 eV, respectively [41]. The band gap of the BiVO_4_@ZnO heterojunction composites lies between those of pure ZnO nanorods and pure BiVO_4_, indicating that the composite structure enables effective band gap modulation. This phenomenon is likely due to interfacial charge transfer effects and the coupling of the energy band structures.

**Figure 7:**
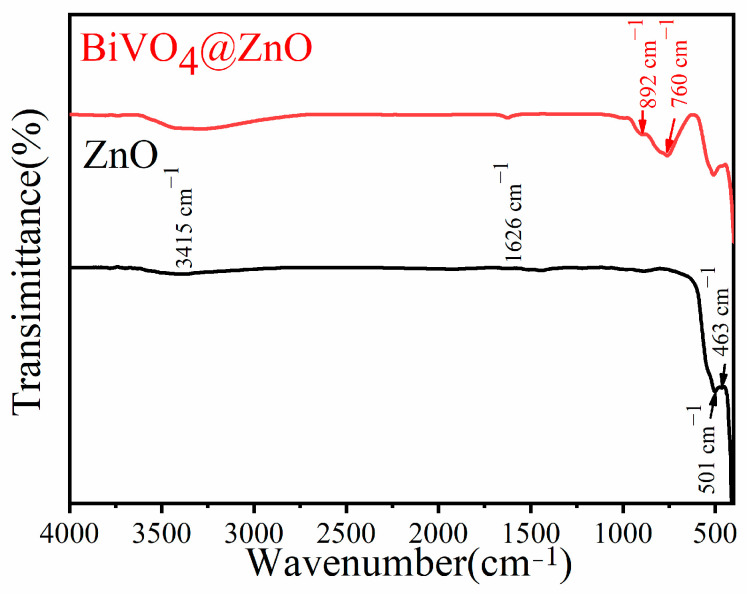
FT-IR spectra of the as-synthesized pure ZnO flower-like nanorods, BiVO_4_@ZnO heterojunction composites.

Figure 7 presents the FT-IR spectra of the pure ZnO flower-like nanorods and the BiVO_4_@ZnO heterojunction composites, which were used to identify the functional groups present in the samples. In the ZnO spectrum, characteristic peaks were observed at 463, 501, 1626, and 3415 cm^−1^. The peak at 1626 cm^−1^ is attributed to the bending vibration of O-H groups and C−O band stretching, while the peak at 3415 cm^−1^ corresponds to the O-H stretching vibration of water molecules adsorbed on the sample surface [42,43]. The absorption peak at 463 cm^−1^ is assigned to the stretching vibration of Zn-O bonds in the synthesized ZnO nanorods [44]. For the BiVO_4_@ZnO heterojunction composites, the broad absorption bands at low frequency (such as 760  and 892 cm^−1^) are attributed to the bending vibration of the VO_4_^3−^ tetrahedron [45]. These observations confirm the coexistence of both ZnO and BiVO_4_ in the nanocomposite, a conclusion that is consistent with the results from X-ray diffraction (XRD) analysis.

**Figure 8:**
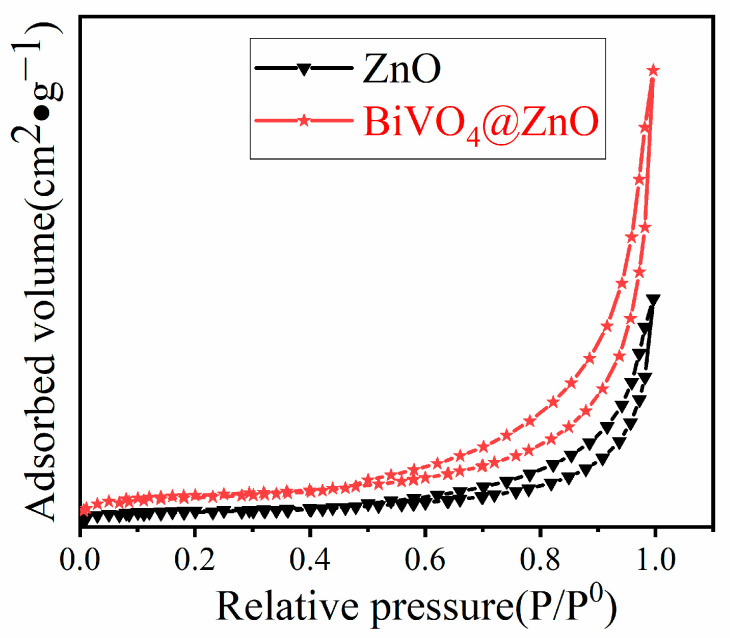
Adsorption–desorption isotherms of the as-synthesized pure ZnO flower-like nanorods, BiVO_4_@ZnO heterojunction composites.

The surface properties and pore structures of pure ZnO flower-like nanorods and BiVO_4_@ZnO heterojunction composites were investigated using the N_2_ adsorption–desorption method, as shown in Figure 8. It can be seen from Figure 8 that the N_2_ adsorption–desorption isotherms of both the ZnO flower-like nanorods and the BiVO_4_@ZnO heterojunction composites exhibit typical type IV isotherm characteristics, indicating that both materials have mesoporous structures. According to the data in Table 1, the BET specific surface area of BiVO_4_@ZnO heterojunction composites is 7.8308 m^2^/g, which is significantly higher than that of the pure ZnO flower-like nanorods (4.8432 m^2^/g). Notably, the specific surface area of the BiVO_4_@ZnO heterojunction composites is markedly increased. This can be attributed to the formation of hierarchical pore structures during the construction of the heterostructures. The larger specific surface area can provide more abundant active sites for photocatalytic reactions, thereby enhancing the efficiency of the photocatalytic process.

**Figure 9:**
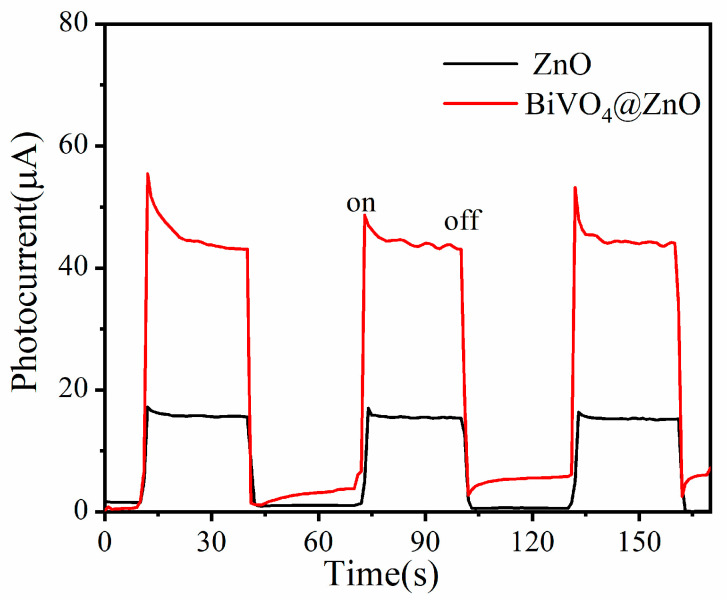
Transient photocurrent of adsorption–desorption isotherms of the as-synthesized pure ZnO flower-like nanorodsa nd BiVO_4_@ZnO heterojunction composites.

To gain deeper insight into the separation and transfer behavior of photogenerated charge carriers, transient photocurrent measurements were conducted on the as-prepared pure ZnO flower-like nanorods and BiVO_4_@ZnO heterojunction composites (as shown in Figure 9) under intermittent light irradiation with a 30 s on/off cycles. The results reveal that both materials exhibit a rapid increase in photocurrent upon illumination, which almost completely decays to zero once the light is switched off. Specifically, the steady-state photocurrent density of the pure ZnO nanorods is approximately 15.7 μA·cm^−2^, whereas that of the BiVO_4_@ZnO heterojunction composites is significantly enhanced to about 43.3 μA·cm^−2^, nearly four times higher. This pronounced improvement indicates that the construction of the p-n heterojunction effectively promotes the generation and separation of photogenerated electron-hole pairs, thereby markedly enhancing carrier transport efficiency and further confirming the excellent photocatalytic performance of the BiVO_4_@ZnO heterojunction composites.

**Figure 10:**
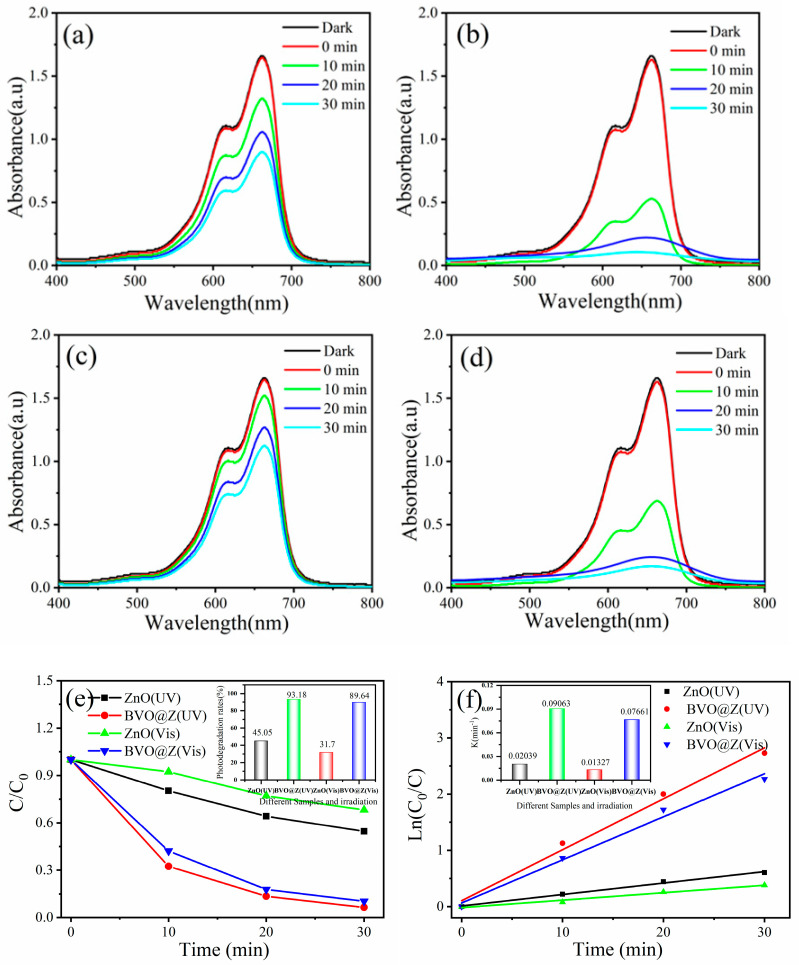
The ultraviolet and visible light photocatalytic performance of the pure ZnO flower-like nanorods and BiVO_4_@ZnO heterojunction composites toward the MB of dye. The absorbance spectra of the MB dye aqueous solution taken at interval time under the ultraviolet and visible light in the presence of (**a**,**c**) pure ZnO flower-like nanorods, (**b**–**d**) BiVO_4_@ZnO heterojunction composites. (**e**) Photodegradation and (**f**) the pseudo-first-order kinetics investigation of pure ZnO flower-like nanorods structures materials and BiVO_4_@ZnO heterojunction composites under MB dye aqueous solution.

The photocatalytic activity of pure ZnO flower-like nanorods and BiVO_4_@ZnO heterojunction composites was quantitatively evaluated by measuring the decomposition of an aqueous MB solution under ultraviolet and visible light over irradiation durations ranging from 0 min to 30 min. The decrease in MB concentration was monitored at a wavelength of 675 nm using a UV-Vis spectrophotometer, as shown in Figure 10. Figure 10a,b show the concentration changes over time during the photocatalytic degradation of MB under UV light irradiation for pure ZnO flower-like nanorods and BiVO_4_@ZnO heterojunction composites, respectively. Similarly, Figure 10c,d illustrates the corresponding concentration changes under visible light (Vis) irradiation. After 30 min of continuous exposure to either UV or visible light, both the pure ZnO flower-like nanorods and the BiVO_4_@ZnO composites effectively reduced the main absorption peak of MB. However, the decrease was more pronounced for the BiVO_4_@ZnO composites, which exhibited a significantly higher degradation rate compared to the pure ZnO catalysts. The photodegradation percentages for pure ZnO under ultraviolet and visible light were calculated to be 45.05% and 31.70%, respectively, indicating better performance under UV light. Its degradation efficiency is considerably limited in the visible region due to its wide band gap. In contrast, the BiVO_4_@ZnO heterojunction composites achieved photodegradation percentages of 93.18% and 89.64% under ultraviolet and visible light, respectively—significantly higher than those of the pure ZnO photocatalysts. This notable enhancement can be attributed to the introduction of BiVO_4_, which not only extends the light absorption into the visible region but also promotes efficient separation and migration of photogenerated charge carriers through interfacial effects, thereby greatly improving the photocatalytic activity.

The photocatalytic activity of these dyes can be described using the Langmuir–Hinshelwood (L–H) kinetic model [46], which is a mechanism where surface reactions are the rate-controlling steps and involve two adsorbed molecules reacting on a solid catalyst surface:

$$\ln(C_{0}/C)=kt$$

In the L–H kinetic model, *k* represents the rate constant, *C*_0_ is the initial dye concentration at absorption-desorption equilibrium (*t* = 0), and *C* is the dye concentration at time *t*. The rate constants *k* for the photocatalytic degradation of MB dye using pure ZnO flower-like nanorods under UV and visible light are 0.02039 and 0.01327, respectively, while those for the BiVO_4_@ZnO heterojunction composites are 0.09063 and 0.07661, respectively. A comparison of these *k* values shows that the BiVO_4_@ZnO composite exhibit higher rate constants than the pure ZnO nanorods, which is consistent with the temporal concentration changes. Therefore, it can be concluded that the BiVO_4_@ZnO heterojunction composites significantly enhance the photocatalytic degradation capability.

**Figure 11:**
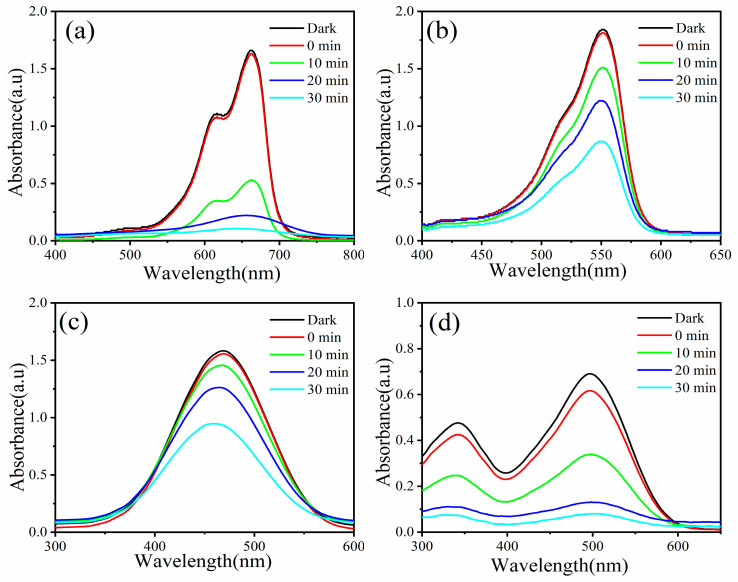

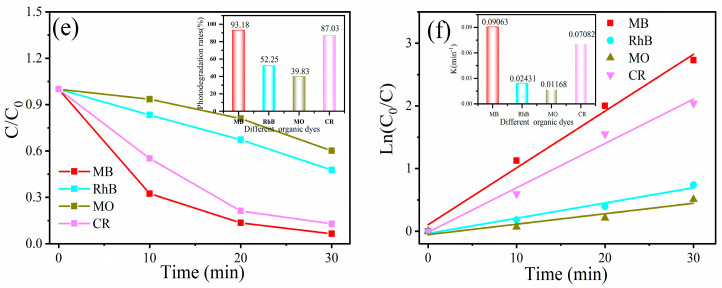
The ultraviolet light photocatalytic performance of BiVO_4_@ZnO heterojunction composites toward the four types of dye. The absorbance spectra of the solutions taken at interval time toward (**a**) MB, (**b**) RhB, (**c**) MO, and (**d**) CR. (**e**) Photodegradation and (**f**) the pseudo-first-order kinetics investigation of BiVO_4_@ZnO heterojunction composites under four types of dye aqueous solution.

The photocatalytic performance of BiVO_4_@ZnO heterojunction composites towards the common organic dyes, including methylene blue (MB), rhodamine B (RhB), methyl orange (MO), and congo red (CR), under ultraviolet light irradiation is shown in Figure 11. The decrease in the concentrations of MB, RhB, MO, and CR was monitored over time by measuring absorbance changes at their characteristic wavelengths of 675 nm, 552 nm, 465 nm, and 495 nm, respectively. The results indicate that the main absorption peaks of these organic dyes decreased significantly within 30 min under UV light irradiation in the presence of BiVO_4_@ZnO composites (see Figure 11a–d). This demonstrates that the BiVO_4_@ZnO heterojunction composites exhibit highly selective degradation performance toward MB dye in aqueous solution. As shown in Figure 11f, the rate constants k for the degradation of MB, RhB, MO, and CR by BiVO_4_@ZnO heterojunction composites are 0.09063, 0.02431, 0.01668, and 0.07082, respectively. From the results in Figure 10f and Figure 11e, it becomes evident that the degradation selectivity of BiVO_4_@ZnO heterojunction composites for MB (methylene blue) stems not from a single factor, but from a complex interplay: the synergistic effects of electrostatic interaction, molecular structure differences, and adsorption behavior [47,48,49,50]. Compared to RhB (rhodamine B), MO (methyl orange), and CR (Congo red), MB, as a cationic dye, readily forms electrostatic attraction with the typically negatively charged catalyst surface, whereas MO and CR, being anionic dyes, face electrostatic repulsion. Furthermore, MB’s compact molecular dimensions (1.2 × 0.8 nm) are smaller than those of RhB (1.8 × 1.0 nm) and CR (2.2 × 1.1 nm), allowing it to penetrate the catalyst’s pore channels and access active sites more effectively. Crucially, MB’s key functional group, -N(CH_3_)_2_ (amino group), forms robust coordination bonds with active sites. This contrasts sharply with RhB’s weakly coordinating -COOH group and the -SO_3_^−^ groups of MO and CR, whose electron-withdrawing nature impedes degradation. At the adsorption level, MB overshadows the others: its saturated adsorption capacity and chemical adsorption strength on the catalyst significantly exceed the loose physical adsorption exhibited by RhB and the extremely low adsorption capacity shown by MO/CR. This superior adsorption provides ample “raw materials” and ensures stable interfacial contact for degradation, ultimately yielding a markedly higher degradation kinetic constant for MB compared to the other three dyes.

**Figure 12:**
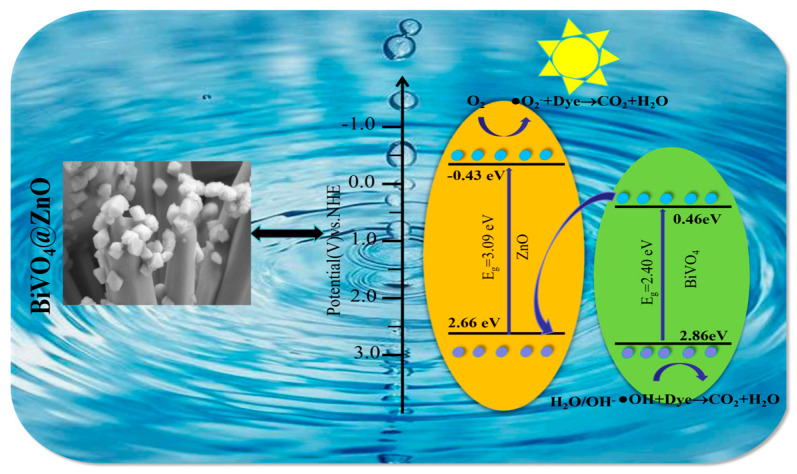
Schematic diagram of degradation mechanism of BiVO_4_@ZnO heterojunction composites in dye solution.

As illustrated in Figure 12, the band positions of BiVO_4_ and ZnO suggest that the conventional Type-II heterojunction model fails to align with the redox reaction sites depicted in the figure, whereas the S-scheme mechanism offers a consistent explanation for this band configuration. The conduction band (CB) and valence band (VB) of ZnO are situated at –0.34 eV and +2.66 eV, respectively, yielding a band gap (Eg) of 3.09 eV. Conversely, the conduction and valence bands of BiVO_4_ lie at +0.46 eV and +2.86 eV, respectively, corresponding to a band gap of 2.40 eV [51,52]. Additionally, the formation potential of •OH/OH^−^ or •OH/H_2_O is approximately +2.4 eV.

Under light irradiation, both ZnO and BiVO_4_ generate electron–hole pairs. In BiVO_4_@ZnO heterojunction composites, the conduction band potential of ZnO (−0.43 eV) lies below that of BiVO_4_ (+0.46 eV); consequently, photogenerated electrons in the conduction band of BiVO_4_ cannot be transferred to the conduction band of ZnO [53,54]. However, the photogenerated electrons in the conduction band of BiVO_4_ can recombine with the photogenerated holes in the valence band of ZnO through a new charge transfer pathway [55]. As a result, the photogenerated holes remain in the BiVO_4_ semiconductor, and their strong oxidizing ability directly participates in oxidation reactions, oxidizing water molecules into •OH (h^+^ + OH^−^→ •OH). As a strong oxidant, •OH can further oxidize and decompose organic pollutants(•OH + Dye → CO_2_ + H_2_O) [56]. Meanwhile, the photogenerated electrons in the conduction band of ZnO are retained, which avoids the direct recombination of electrons and holes. These electrons further react with dissolved oxygen to generate superoxide radicals (O_2_ + e^−^ → •O_2_^−^), thereby achieving the degradation of pollutants (•O_2_^−^ + Dye → CO_2_ + H_2_O) [57]. This S-scheme heterojunction structure enables the efficient transfer of photogenerated electrons and holes, concentrating the photogenerated charge carriers in different semiconductors respectively and reducing the recombination of photogenerated electron-hole pairs. At the same time, the oxidizing ability of holes and the reducing ability of electrons are preserved, which is more conducive to efficient photocatalytic reactions. This endows the BiVO_4_@ZnO heterojunction with better performance in reactions such as photocatalytic degradation of organic pollutants. Therefore, Z-scheme heterojunctions are widely applied in fields like photocatalytic pollutant degradation and serve as one of the important strategies for constructing high-efficiency photocatalysts.

**Figure 13:**
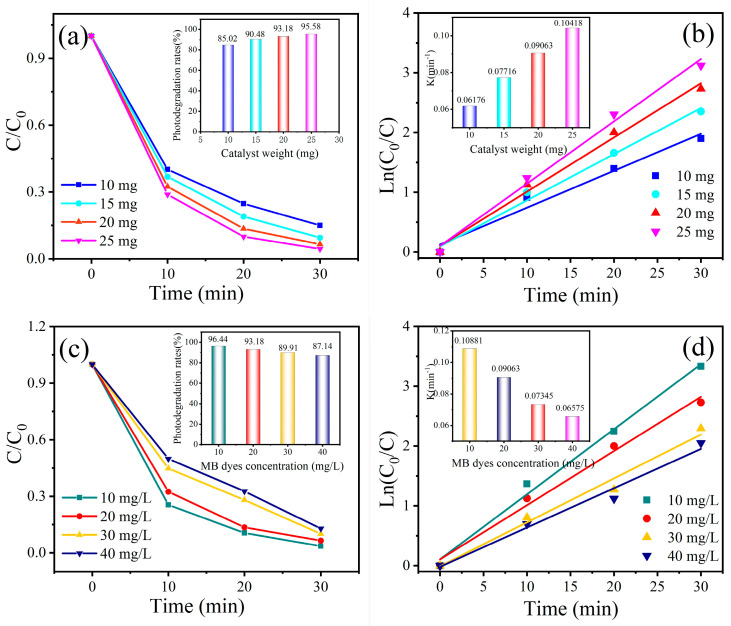
The ultraviolet photocatalytic performance of the BiVO_4_@ZnO heterojunction composites toward the MB of dye. (**a**) Photodegradation and (**b**) the pseudo-first-order kinetics investigation of BiVO_4_@ZnO heterojunction composites with different weights under MB dye aqueous solution. (**c**) Photodegradation and (**d**) the pseudo-first-order kinetics investigation of BiVO_4_@ZnO heterojunction composites under different concentrations of MB dye aqueous solution.

To investigate the effects of catalyst weight and initial concentration of methylene blue (MB) dye on photocatalytic degradation performance, time-dependent degradation curves and corresponding pseudo-first-order kinetic analyses were conducted, as shown in Figure 13a–d. Figure 13a,b shows the effect of the weight of the BiVO_4_@ZnO heterojunction composites on the degradation efficiency of MB dye solution. When the weight of BiVO4@ZnO heterojunction composites increases from 10 mg to 25 mg, the degradation efficiency of MB dye solution rises from 85.02% to 95.58%, and the pseudo-first-order rate constant k increases from 0.06176 min^−1^ to 0.10418 min^−1^. This improvement can be attributed to the increase in the number of active sites available for photocatalytic reactions after the weight of BiVO_4_@ZnO heterojunction composites catalyst increases. Figure 13c,d illustrates the effect of the initial concentration of MB dye solution on the photocatalytic performance of BiVO_4_@ZnO heterojunction composites. When the initial concentration of MB dye increases from 10 mg/L to 40 mg/L, the degradation efficiency of MB dye solution by BiVO_4_@ZnO heterojunction composites decreases from 96.44% to 87.14%, and the k value drops from 0.10881 min^−1^ to 0.06575 min^−1^. The phenomenon of the degradation efficiency of BiVO_4_@ZnO heterojunction composites decreasing at higher MB dye concentrations may be associated with the saturation of active sites on the catalyst surface, where excessive MB molecules would limit light absorption and mass transfer during the photocatalytic process.

**Figure 14:**
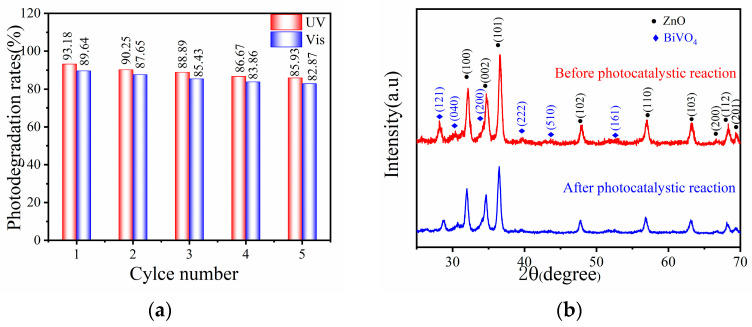
(**a**) Photocatalytic recyclability test of BiVO_4_@ZnO heterojunction composites in MB dye aqueous solution under ultraviolet and visible light, and (**b**) XRD patterns of the BiVO_4_@ZnO heterojunction composite materials before and after the cyclic photocatalytic degradation test.

The photocatalytic cycling stability of the BiVO_4_@ZnO heterojunction composites toward an aqueous MB dye solution under ultraviolet and visible light is shown in Figure 14a. After five degradation cycles, the photocatalytic degradation efficiency of the samples for the MB solution remains above 80%, indicating that the BiVO_4_@ZnO heterojunction composites exhibit excellent cyclic catalytic stability. Furthermore, XRD characterization of the BiVO_4_@ZnO heterojunction composites before and after cyclic photocatalysis (Figure 14b) reveals that the positions of the characteristic diffraction peaks of ZnO and BiVO_4_ in the composite show no obvious shift, with only a slight decrease in peak intensity. This phenomenon can be attributed to the adsorption of trace reaction intermediates on the material surface during photocatalysis, rather than substantial destruction of the crystal structure, further confirming the structural stability of the composite in photocatalytic reactions. The above results demonstrate that the BiVO_4_@ZnO heterojunction composites not only exhibit high-efficiency MB degradation performance but also possesses excellent stability and environmental compatibility during repeated use, providing important support for its practical application in water treatment.

## 4. Conclusions

In summary, this study successfully synthesized a highly efficient BiVO_4_@ZnO heterojunction composite through a two-step hydrothermal method for the degradation of organic dyes. By integrating polyhedral BiVO_4_ with flower-like ZnO nanostructures, the composite demonstrates an enlarged specific surface area, extended light-response range, and improved separation of photogenerated charge carriers. Under UV irradiation, the catalyst achieved degradation rates of 93.18% for MB and 87.03% for CR within 30 min, while under visible light, it also attained a high degradation efficiency of 89.64% for MB within the same period. Moreover, the material exhibited excellent cycling stability, maintaining over 80% degradation efficiency after five consecutive cycles, as well as structural stability with no significant shift in XRD diffraction peaks, indicating strong potential for practical applications. The primary contribution of this work lies in the design and construction of a novel Type-II heterojunction photocatalyst, which facilitates efficient charge separation and catalytic degradation under broad-spectrum light irradiation. This study systematically elucidates the structure–performance relationship of the composite, offering valuable insights into the development of efficient and stable solar-driven materials for environmental remediation. The material demonstrates promising application prospects in various fields, particularly in the advanced treatment of organic pollutants in industrial wastewater, the development of visible-light-driven photocatalytic water purification systems, and the promotion of sustainable environmental remediation technologies. It provides both a new material platform and technical support to advance the practical implementation of photocatalytic technology.

## Figures and Tables

**Table 1:** BET surface area of the as-synthesized pure ZnO flower-like nanorods, BiVO_4_@ZnO heterojunction composites.

Sample	BET Surface Area, (m^2^/g)
ZnO	4.8432
BiVO_4_@ZnO	7.8308

## Data Availability

The original contributions presented in the study are included in the article, and further inquiries can be directed to the corresponding author.
